# Chronic Abruptio Placentae With Multiple Alloantibodies: An Obstetrician's Challenge

**DOI:** 10.7759/cureus.47762

**Published:** 2023-10-26

**Authors:** Girija S Mohanty, Tanvi Katoch, Sujata Siwatch, Divjot S Lamba, Ratti R Sharma

**Affiliations:** 1 Obstetrics and Gynaecology, Postgraduate Institute of Medical Education and Research, Chandigarh, IND; 2 Transfusion Medicine, Postgraduate Institute of Medical Education and Research, Chandigarh, IND

**Keywords:** antepartum hemorrhage, incompatible blood, maternal alloimmunization, placental separation, rh alloimmunization, chronic abruption

## Abstract

The current case highlights the management of abruptio placentae in pregnant women with an O Rhesus (Rh)-negative blood group with multiple alloantibodies. We describe a unique case of chronic placental separation in a young primigravida presenting with intrauterine hematoma and intrauterine fetal death (IUFD), who had an O Rhesus-negative blood group with alloimmunization against D, C, and S antigens. The implications in management were the dilemma in diagnosis, the ABO blood grouping discrepancy, multiple alloantibodies including Rh alloimmunization, chronic placental abruption with postpartum hemorrhage, and scope for further pregnancies. Chronic placental separation or abruption can occur silently in some cases. On presentation, they may be mistaken with or for other lesions. In Rh-negative pregnancies, chronic abruption can lead to alloimmunization against Rh and other clinically significant antigens as well. Women with suspicion for chronic abruption must undergo detailed blood group testing as well as immunohematological workup at a nearby transfusion medicine department with a facility for complex immunohematological resolutions.

## Introduction

Abruptio placentae is one of the major causes of maternal and perinatal mortality and morbidity. Ten percent of abruption is associated with clinically significant coagulopathies [1]. Abruption is characterized by retroplacental hemorrhage due to bleeding in the basal layer of the decidua. The precise initiating event or cause of such a bleed is still elusive. The vessels could be pathologically altered and become prone to rupture. There could be vascular malformations leading to rupture [2]. Management of placental abruption is a challenging situation for the obstetrician. When concealed or chronic, it may mimic placental tumors, uterine rupture, and adnexal masses. Abruption is a risk factor for Rhesus (Rh) alloimmunization in women with Rh-negative blood group [2].

## Case presentation

**Figure 1:**
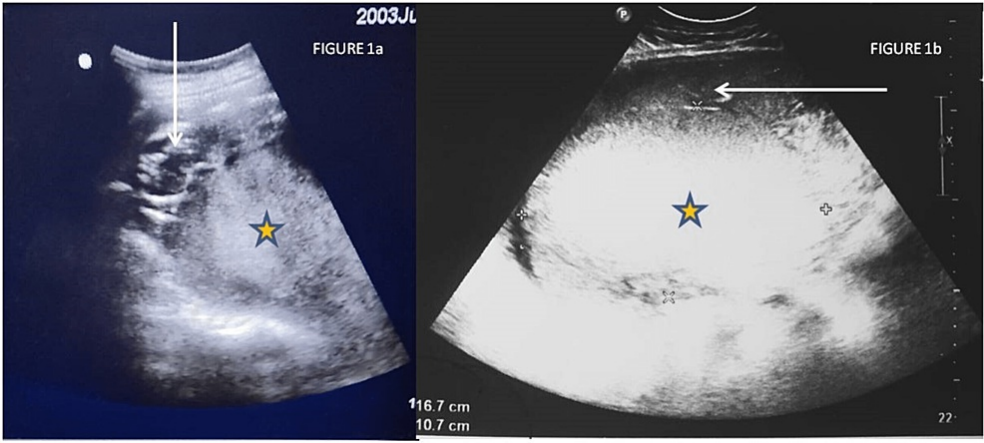
1a: Transabdominal ultrasound showing the placenta (arrow) pushed anteriorly by the mass (star). 1b: Transabdominal ultrasound showing the intrauterine homogeneous mass measuring 16.7 × 10.7 cm (star) in relation to the placenta (arrow).

A 22-year-old primigravida in the sixth month of pregnancy was referred to our center from a rural area with sonographic evidence of a large heterogeneous mass occupying the whole abdomen and a single dead fetus seen in the right hypochondrium with parameters corresponding to 25 weeks. The patient had a low body mass index and low socioeconomic status. There was no history of hypertension, smoking, infection, trauma, or preterm rupture of membranes. Her pregnancy was not well supervised, and she had not perceived quickening yet. She vaguely gave a history of minimal vaginal bleeding on and off. She had abdominal distension gradually for the last 20 days, and it was painless, appearing as normal pregnancy growth to her family. She had severe pain one day ago when she sought medical care, and the above-referring ultrasound was done. A previous scan done three months prior at a peripheral center showed a crown-rump length corresponding to 12 weeks and five days with cardiac activity present and the placenta developing posteriorly. On admission, her blood pressure was 90/50 mmHg, her pulse was 130 per minute, her hemoglobin was 6.1 g/dL, and her renal function was maintained with creatinine of 0.7 mg/dL. The abdomen was tense and tender, with a fundal height of 32 weeks, and the flanks were full. On per vaginal examination, the cervical os was closed and uneffaced. On ultrasound at our center, uterine myometrium looked distended with a large homogeneous lesion, likely a large clot or organized blood of 16 × 11 cm, which seemed to have pushed the placenta anteriorly and the fetus toward the right hypochondrium; however, the uterine contour was maintained (Figure 1a, 1b).

**Figure 2:**
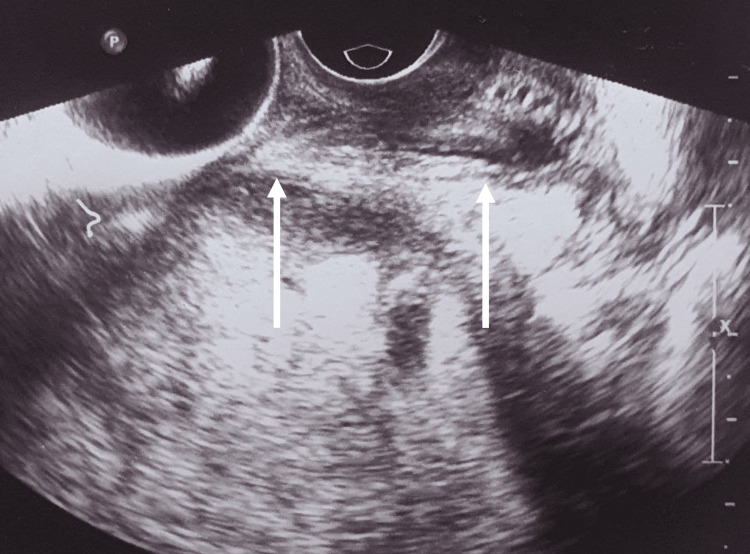
Transvaginal ultrasound showing the cervix (arrows) with closed internal and external os.

The cervix was almost 3 cm long on transvaginal ultrasound, and the internal os was closed (Figure 2).

A working diagnosis of chronic abruption was kept, with a differential diagnosis of intrauterine mass and uterine rupture. The patient was planned for laparotomy with hysterotomy, and a blood requisition was sent to arrange blood for the surgery. The patient's blood group was O RhD-negative on routine blood grouping by conventional tube technique (cell grouping for ABO and RhD). On serum grouping with pooled ABO cells, she developed 3+ agglutination with pooled O cells indicating ABO discrepancy, whereas 4+ reaction with A cells and B cells collaborated with the O group in forward grouping. Reaction with anti-H lectin was positive, indicating O blood group only. The patient did not give any history of previous transfusion in the past. Due to the blood group discrepancy and due to crossmatch incompatibility between donor and patient samples with multiple donor packed red blood cell (PRBC) units, the case was referred to the immunohematology laboratory of the department.

The direct antiglobulin test (DAT) and auto-control (AC) were negative on polyspecific anti-human globulin (AHG) gel cards (LISS-Coombs AHG gel card, Bio-Rad, Switzerland) based on column agglutination technique (CAT). An antibody screen using a three-cell panel (DiaCell I-II-III, Bio-Rad, Switzerland) was done and found to be positive, which was followed by antibody identification done by CAT using an 11-cell panel (DiaPanel, Bio-Rad, Switzerland), and the presence of anti-D, anti-C, and anti-S antibodies was confirmed in the results obtained. Titration for these antibodies could not be done due to time constraints. The presence of these antibodies collaborated with extra reaction obtained in reverse grouping with pooled O cells. The dosage phenomenon was also observed for anti-S antibodies, as the strength of the reactions was stronger in the cells carrying a double dose of S antigens compared to heterozygous cells, i.e., S+s+. Fifteen PRBC O RhD-negative units were typed out, of which only three were found to be both C- and S-negative. Finally, all three units of PRBC were found to be least incompatible (1+) by the column agglutination technique (CAT) [3]. The transfusion medicine department released these units after taking the least incompatible consent for transfusion to the patient during lifesaving surgery. The need for the urgent transfusion and lifesaving surgery was explained to the attendants. On laparotomy, the uterus was distended, and there was no free fluid or blood in the abdomen. On incising the lower segment of the uterus, approximately 2 liters of altered old blood with the whole placenta expelled on its own, following which the fetus was extracted. The uterus was arcuately shaped, and bilateral fallopian tubes and ovaries were normal in morphology. The uterus was atonic, so after medical management with oxytocic, bilateral uterine artery ligation was done, and Hayman sutures were applied after compressing the uterus manually. She was transfused two units of least incompatible PRBC and one unit of platelet concentrate intraoperatively. The weight of the stillborn baby was 570 grams, and it had darkened slipping skin suggestive of old intrauterine fetal demise (IUFD). She was transferred to the intensive care unit for supportive care and was given broad-spectrum antibiotics. Postoperatively, she improved, and her hemoglobin post-transfusion was 8.4 g/dL. She was discharged on day 5 postoperatively in stable condition.

## Discussion

Placental abruption affects 0.4%-1% of pregnant women [4]. The definition according to the International Classification of Diseases 10th Revision (ICD-10) is "the separation of the placenta from the maternal uterine attachment when it occurs after the 20th week of pregnancy" [5]. Abruption is revealed when it is visible to the eye as bleeding per vagina; otherwise, it is concealed when the hemorrhage gets collected behind the placenta [6]. Concealed abruption may occur in 11%-20% of cases; our case had concealed abruption [7]. The etiology of abruption is not known well and is hypothesized to be a part of a wider placental syndrome involving vasculopathy and defective placentation [8]. Factors that lead to the above syndrome chronically include maternal vasculopathy, hypertension (chronic or preeclampsia), smoking, intake of drugs, nutritional deficiency, uterine malformations and tumors, supine hypotension syndrome, antiphospholipid syndrome, and thrombophilias. As in our patient, no identifiable cause is found in more than 40% of cases [9].

The most common presentation of abruption is the triad of abdominal pain, abnormal uterine tenderness, and bleeding per vagina, or any of these symptoms may be present. Almost 18%-20% present with no clinical symptoms [6]. Our patient presented with abdominal pain. However, the vague abdominal distension, which she mistook for pregnancy-related distension, was also caused by abruption gradually over time. This was atypical of our case, as it was a chronic abruption. Another evidence of chronic abruption in our case was the patient's hemodynamic stability and absence of coagulopathy, despite the abruption of 2 liters of hemorrhage.

The diagnosis of placental abruption is made by evidence of antepartum hemorrhage or can be made with ultrasonography in cases of concealed abruption [10]. This case had a diagnostic dilemma due to a large lesion on ultrasound beneath the placenta, disrupting the fetus' location on an ultrasound. Our differentials on ultrasound were placental abruption, intra- or extrauterine mass, and, less likely, uterine rupture. Also, the dilemma further occurred because there was no vaginal bleeding at all, the cervix was uneffaced, and the os was tightly closed. On sonography, abruption can be confused with or mistaken for other lesions [11]. Similar confusion was present in our case, and the possibility of other lesions had to be considered before laparotomy. This hypo-echogenicity of abruption on sonography in our case also depicts the chronic nature of abruption in the current case. Hence, it was undoubtedly present for over two weeks in this case. The hypothesis that explains some of the ambiguities is that retroplacental hemorrhages fall into two broad categories: arterial and venous [12,13]. According to this, arterial hemorrhage leads to acute abruption of the placentae, whereas venous hemorrhage leads to marginal and chronic abruption. In the center two-thirds area of the placenta, arterial blood flow and arterial pressure are maximal. Therefore, abruptio placentae are more likely to be centrally located, causing indentation and rupture of the basal plate and leading to rapid delivery often associated with fetal hypoxia [12,13]. Conversely, venous hemorrhages from chronically distended veins remain peripheral or marginal. These peripheral veins, previously known as the marginal sinus, are less well-supported than central veins. Since venous pressure is less than arterial pressure, venous hemorrhages are less destructive and do not necessarily lead to immediate delivery. This hypothesis can explain our case's chronicity and less destructive nature of abruption. Gestational hypertension was less common in chronic abruption (also absent in our case), and more likely, it is associated with second-trimester bleeding (possibly present in our case) [13]. The lack of compatible blood for the case led to a more complex scenario in the middle of the night, as it was incompatible with most of the PRBC bags. Incompatibility can be attributed to alloimmunization against multiple antibodies, which must have occurred due to chronic abruption. The long-term complication is alloimmunization and recurrent abruption.

## Conclusions

The patient is currently doing well and leading a healthy life. She has been advised for pre-conceptional counseling before planning a future pregnancy, early and proper pregnancy supervision for monitoring of peak systolic velocity of the middle cerebral artery, and planning intrauterine transfusions if required, as well as detailed immunohematological and Rh alloimmunization workup, along with monitoring of antibody titers due to previous alloimmunization, accordingly in the subsequent pregnancy. Chronic placental separation or abruption can occur silently in some cases. On presentation, they may be mistaken with or for other lesions. In Rh-negative pregnancies, chronic abruption can lead to alloimmunization against Rh and other clinically significant antigens as well. Women with suspicion for chronic abruption must undergo detailed blood group testing as well as immunohematological workup at a nearby transfusion medicine department with a facility for complex immunohematological resolutions. Sometimes, due to its indolent course, women may present late with intrauterine fetal death. Therefore, the general population should be educated to get all pregnancies well supervised.
